# Supplementary material for: Cellular population dynamics shape the route to human pluripotency
Source: Nat Commun. 2023 May 17;14:2829. doi: 10.1038/s41467-023-37270-w (PMC10192362; doi:10.1038/s41467-023-37270-w)
Supplement: Supplementary file 1 — Supplementary Information [file 41467_2023_37270_MOESM1_ESM.pdf]

Supplementary Information

Cellular population dynamics shape the route to human pluripotency

Panariello *et al.*

Supplementary Figures 1 - 6

Supplementary Figure 1

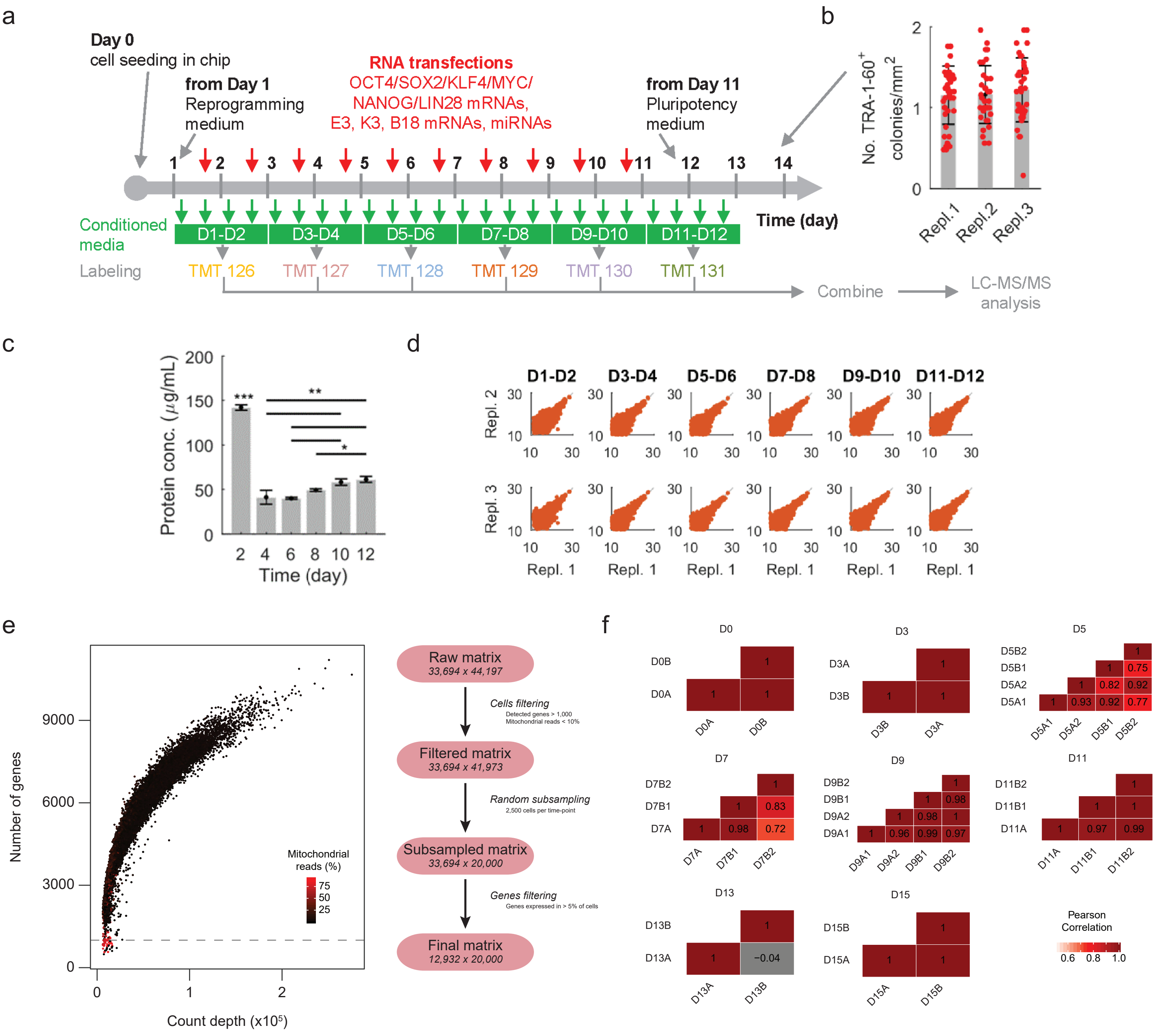

Supplementary Figure 1. Data quality assessment ensures the robustness of the analyses. A) Schematic representation of the experimental design for the proteomic study. B) Reprogramming efficiency within the same microfluidic channels used for proteomic analyses (n=40 for Repl.1, n=35 for Repl.2 and n=39 for Repl.3). Data are presented as mean values +/- SD. C) Results of protein quantification by BCA for samples analyzed by LC-MS/MS. Each value refers to samples obtained by pooling together medium collected from 40 microfluidic channels during 48-hour conditioning (4 time points of collection). Error bar is mean±standard deviation (n=3). Significant differences were evaluated by one-way ANOVA with Tukey's post-test (*p<0.05, **p<0.01, ***p<0.001). D) Visualization of proteomic data correlation between replicates. Each dot represents an identified protein. Log2 relative quantification is shown on the axes (a.u.). E-F) Quality checks of scRNA-seq data. E) Left: Scatter plot representing the number of reads (x axis) over the number of detected genes (y axis) for each cell. Color gradient shows the percentage of reads associated with mitochondrial genes. The dotted line has been put at 1,000 detected genes, used for filtering. Right: Schematic representation of cells/genes filtering from raw data to the final dataset. F) Heatmaps of Pearson correlation coefficient for each replicate, divided by each time-point. Correlation has been evaluated by comparing the distribution of each replicate in the clusters identified in Fig. 3B.

Supplementary Figure 2

a

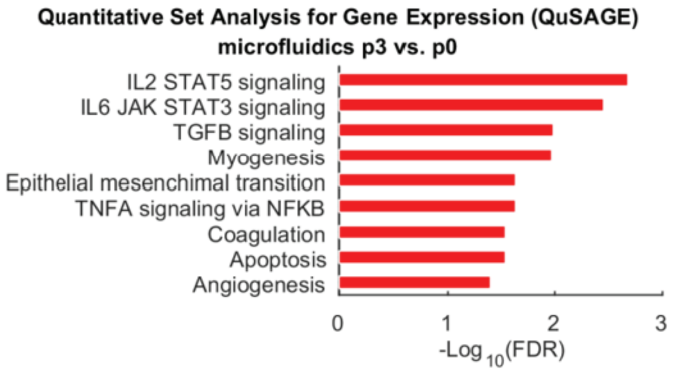

Supplementary Figure 2. JAK-STAT pathway, dowstream of IL6, is activated upon microfluidic-derived hiPSCs culture. A) Analysis of microarray data in Luni et al., 2016[18] by Quantitative Set Analysis for Gene Expression (QuSAGE) within Hallmark gene set in Msig DB collection. Microarray analysis was performed on mRNA extracted from 4 single hiPSC colonies derived in microfluidics (p0) and then expanded for 3 passages in a conventional multi-well plate (p3). Differences show that JAK-STAT pathway is the most significantly different between the two culture systems, suggesting that microfluidic environment may affect this pathway.

Supplementary Figure 3

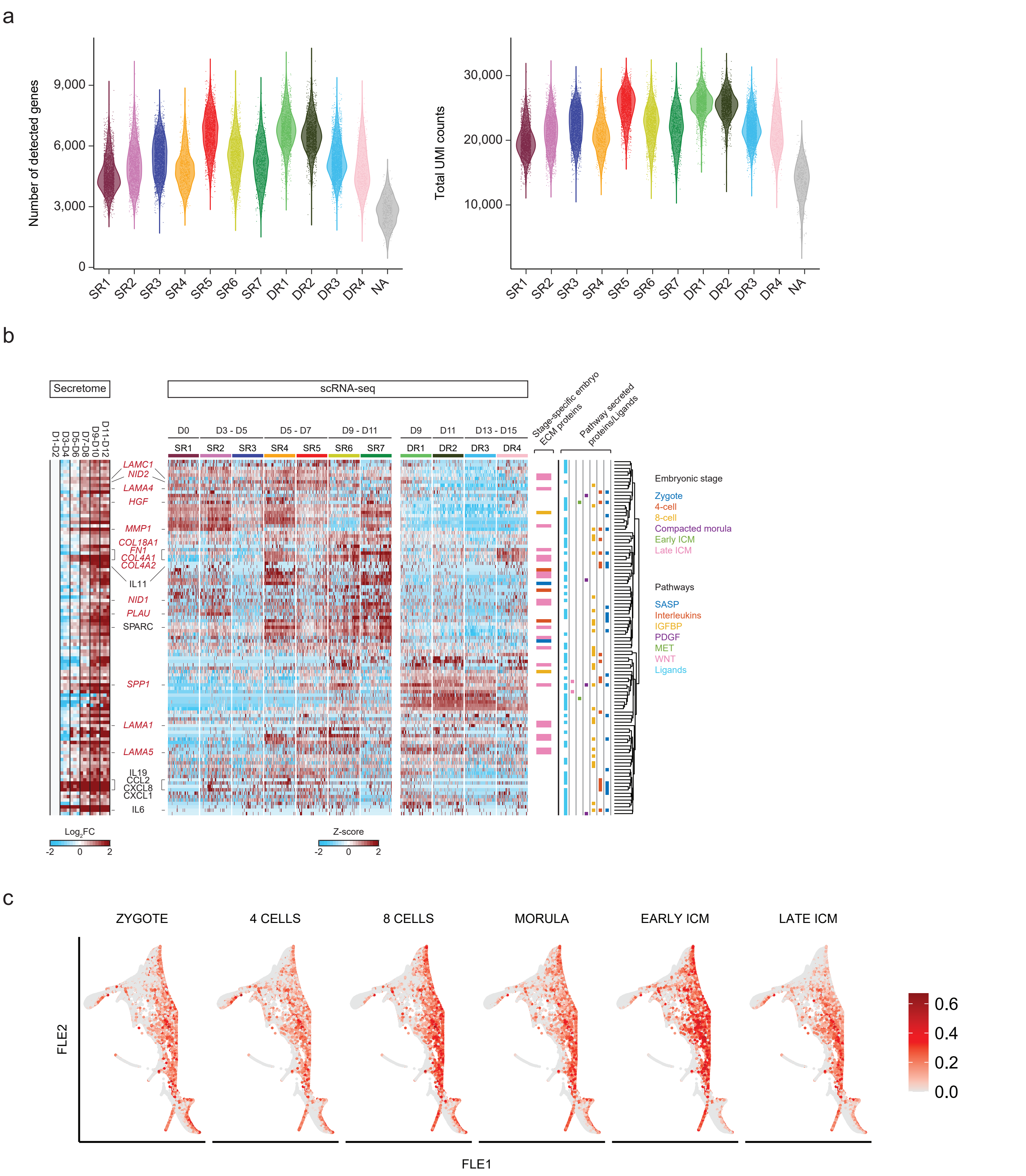

Supplementary Figure 3. SR clusters contribute to the establishment of a permissive extracellular environment. A) Violin plots of the distribution of UMI counts (left) and number of detected genes (right), colored by clusters. B) Heatmaps of highly dynamic proteins from the secretome analysis in Fig. 2B-C are reported. The colors display log2 fold change protein concentration with respect to D1-D2 (Secretome - left) and Z-scored log2 counts per million (scRNA-seq - right). Hierarchical clustering was performed on scRNA-seq data according to each separate cluster of cells. Proteins involved in primitive node formation are highlighted (red names). C) Embryo-related signatures[29] enrichment scores shown along the FLE map.

Supplementary Figure 4

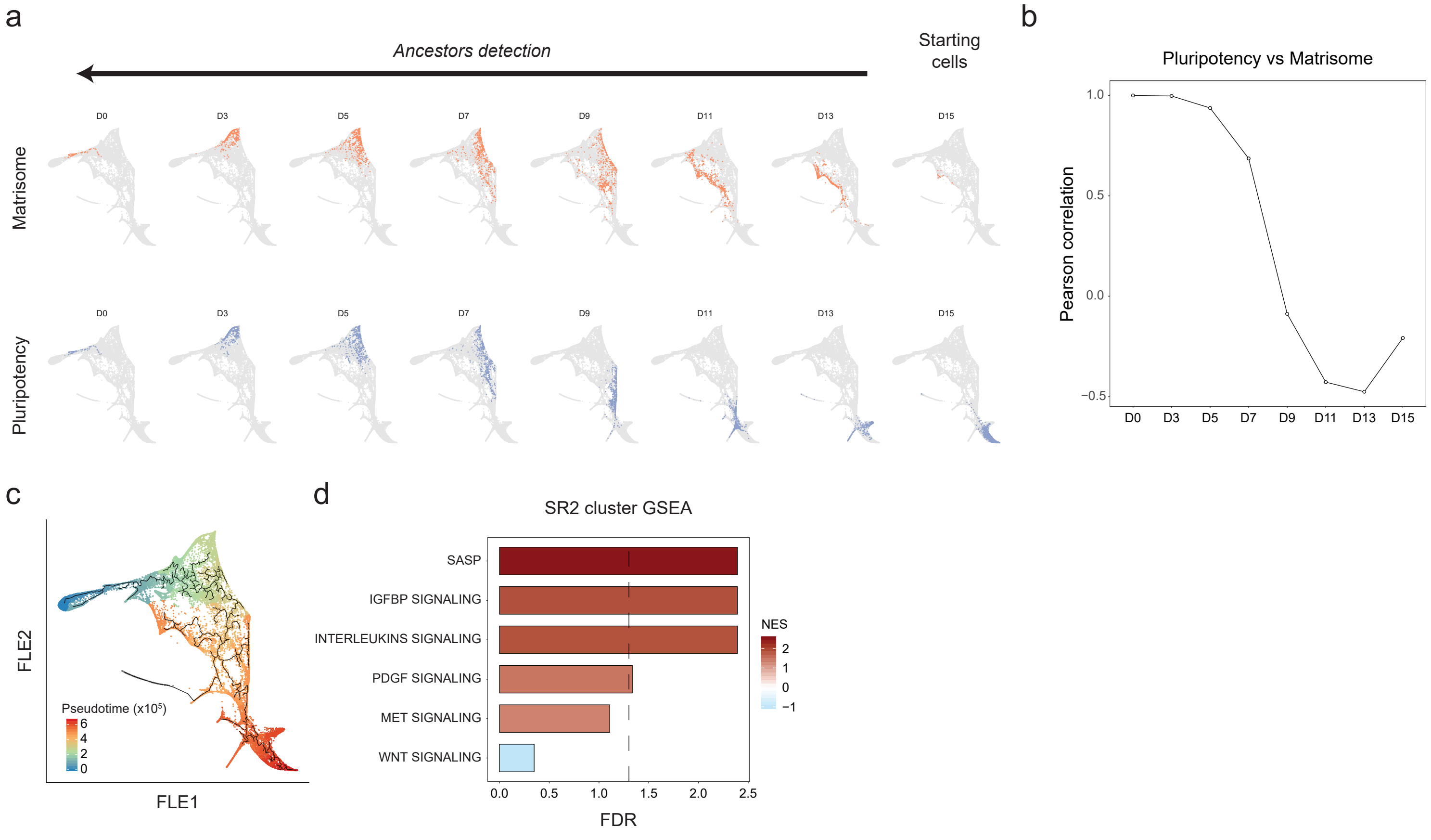

Supplementary Figure 4. Early onset of a SASP-enriched population during reprogramming. A) Workflow for the detection of trajectories starting from Matrisome and Pluripotency D15 enriched cells. Cells assigned to each trajectory are colored and divided by time-point, as described in Methods. B) Trend of pearson correlation coefficients evaluated between trajectories probability values for Matrisome and Pluriptency, assigned to each cell and divided by time-point. C) Monocle3 (black line) trajectory inferences are displayed on the FLE graph. Dots (cells) are colored according to Monocle3 pseudotime. D) GSEA has been performed on SR2 cluster using signaling-related genesets used in Fig. 1F. The results are shown as a barplot, displaying FDR (x axis) and NES (colors). NES, Normalized Enrichment Score. FDR, False Discovery Rate.

Supplementary Figure 5

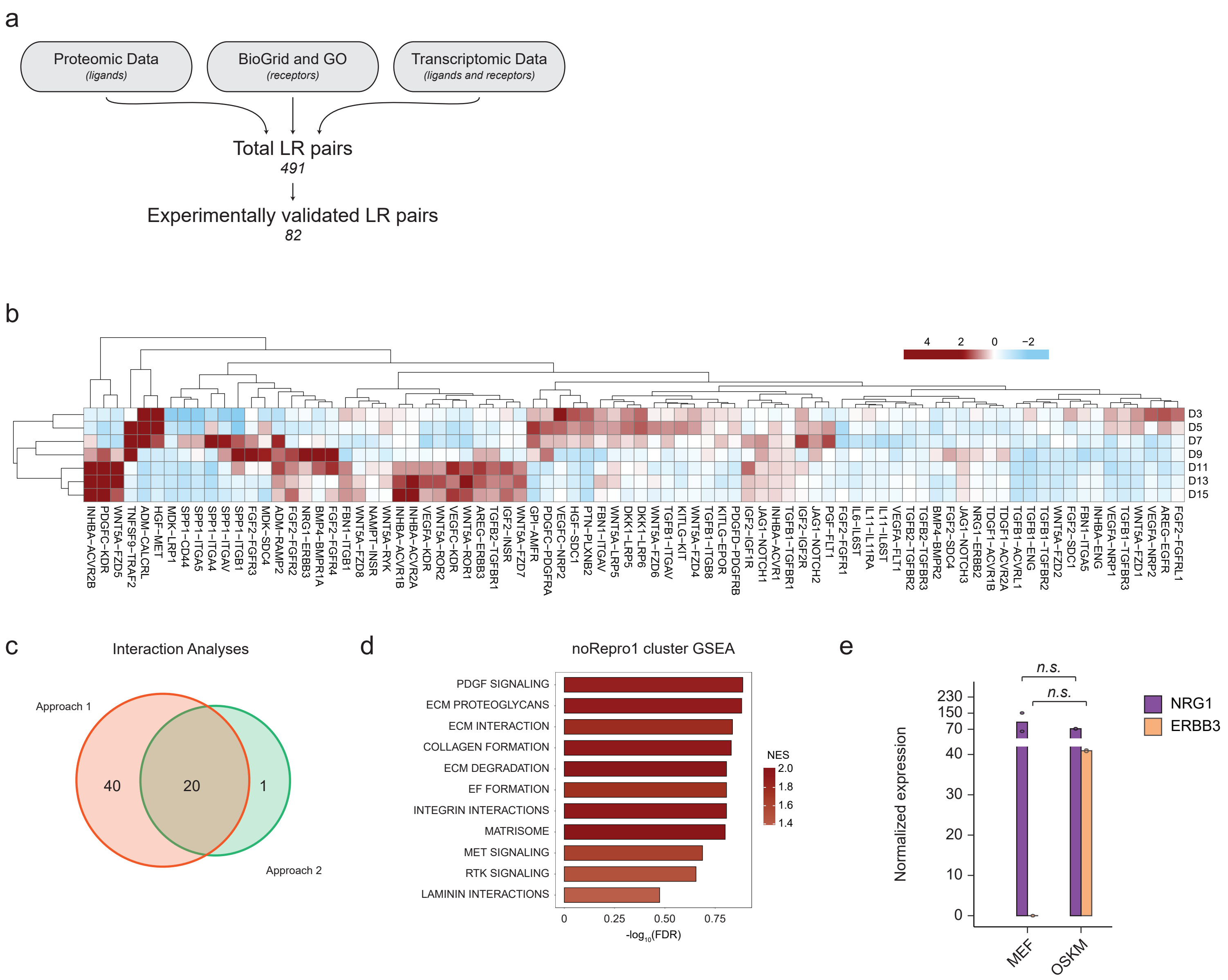

Supplementary Figure 5. Interaction analyses confirm the crosstalk between SR and DR clusters. A) Schematic representation of ligand-receptor pairs selection for interaction score analyses, as described in Methods. B) Heatmap of z-scored standardized interaction scores for all the ligand-receptor pairs analyzed. C) Venn diagram representing the intersection between the number of ligand-receptor pairs with at least one significant interaction score in the reported (orange - Approach 1) or alternative (green - Approach 2) approaches. D) GSEA has been performed on the noRepro1 cluster from Liu et al., 2020[7] using secretome-related genesets used in Fig. 3D right. The results are shown as a barplot, displaying FDR (x axis) and NES (colors). NES, Normalized Enrichment Score. FDR, False Discovery Rate. E) In Cacchiarelli et al., 2015[2], NRG1 and ERBB3 are shown as mouse and human mean normalized expression at sampling day 8 (** BH-adjusted p-value < 0.01).

Supplementary Figure 6

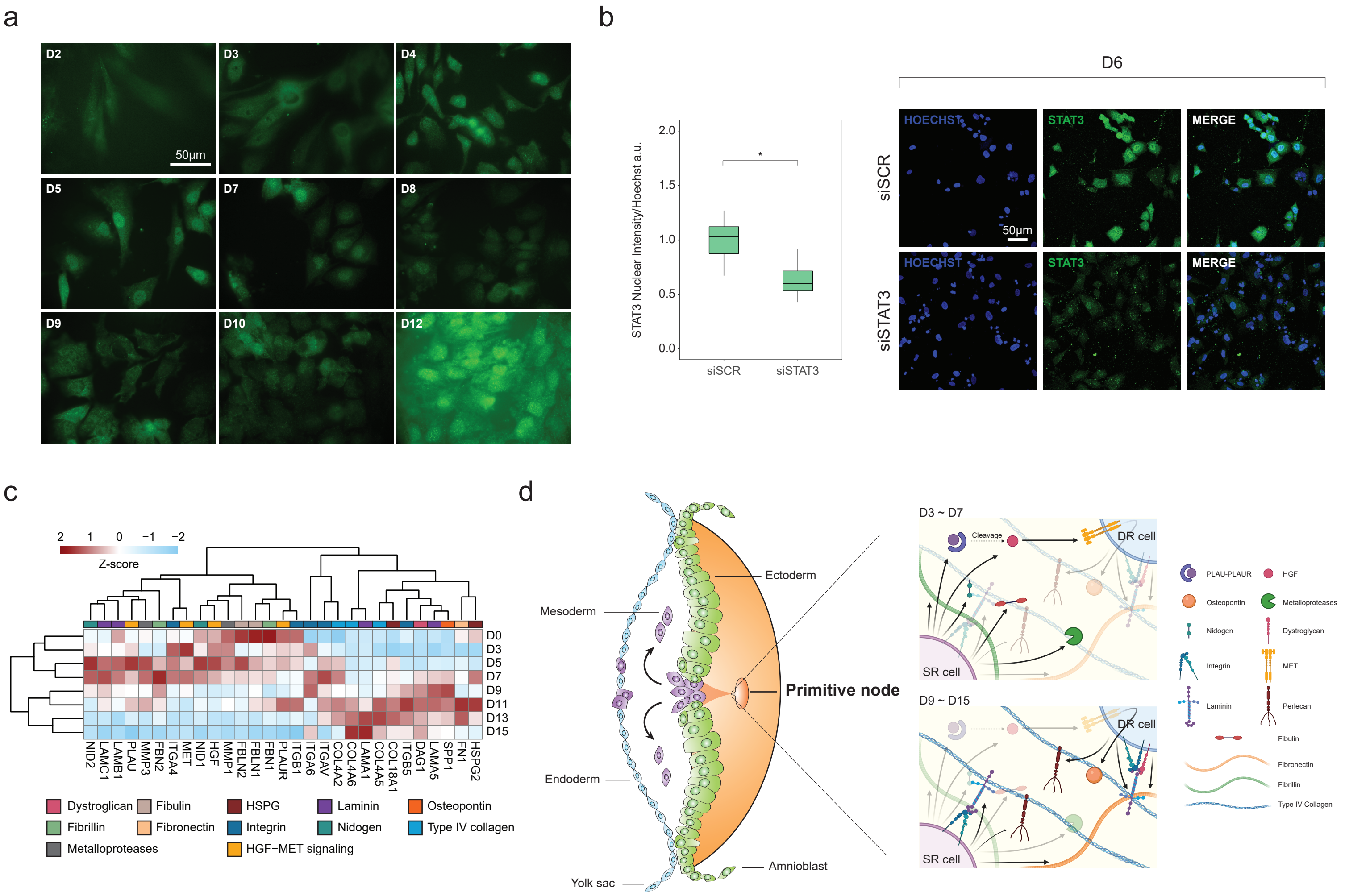

Supplementary Figure 6. HGF/MET physiology during embryo development is recapitulated during reprogramming. A) Time course of STAT3 activation during the microfluidic reprogramming process. Representative images showing a first wave around day 4 to day 7, and then a second wave at the end of the process, when it is active just in the hiPSC colonies. Scale bar, 50 µm. Representative images from n=2 independent experiments. B) Left, quantification of mean STAT3 nuclear intensity versus mean nuclei intensity, stained with Hoechst (n=25 for scramble siRNA, n=37 for siSTAT3); two-sided unpaired t-test was used to assess differences among the conditions (95% CI [-0.4526, -0.3031], * P = 0.0487). Right, representative images. Scale bar, 50 µm. C) Heatmap of Z-scored log2 counts per million, averaged by day, of genes encoding for primitive node components. D) Schematic representation of primitive node formation (left - Adapted from Boccaccio and Comoglio, 2006[1]) and primitive node components (right - Created with BioRender.com). Snapshot of early and late events are reported according to the expression dynamics in (C). Black arrows show the contribution of SR and DR cells based on their average gene expression.

[1] Boccaccio, C., Comoglio, P. Invasive growth: a MET-driven genetic programme for cancer and stem cells. Nat Rev Cancer 6, 637–645 (2006). https://doi.org/10.1038/nrc1912
