# Supplementary material for: Cellular population dynamics shape the route to human pluripotency
Source: Nat Commun. 2023 May 17;14:2829. doi: 10.1038/s41467-023-37270-w (PMC10192362; doi:10.1038/s41467-023-37270-w)
Supplement: Supplementary file 3 — Description of Additional Supplementary Files [file 41467_2023_37270_MOESM3_ESM.pdf]

Description of Additional Supplementary Files

Title: Supplementary Data 1.

Description: Secreted proteins.

Title: Supplementary Data 2.

Description: Results of Reactome enrichment analysis on secreted proteins. Over-representation analysis test was performed. Benjamini-Hochberg adjusted p-values are reported.

Title: Supplementary Data 3.

Description: Data from Figure 2B.

Title: Supplementary Data 4.

Description: Data from Figure 2C.

Title: Supplementary Data 5.

Description: Genesets used to perform GSEA in Figure 3D.

Title: Supplementary Data 6.

Description: Positive and significant results of GSEA on SR2 cluster for Common pathways. GSEA statistical test based on 1000 permutations was used. Benjamini-Hochberg adjusted p-values are reported.

Title: Supplementary Data 7.

Description: Uniquely assigned marker genes divided by cluster.

Title: Supplementary Data 8.

Description: Results from Interaction Score Analysis. Empirical p-value was evaluated by testing 10000 permutations.

Title: Supplementary Data 9.

Description: Data from Figure 5B and Supplementary Figure 5B.

Title: Supplementary Data 10.

Description: Data from Approach 2 in Supplementary Figure 5C. Empirical p-value was evaluated by multiple permutations.
