# Supplementary material for: Cellular population dynamics shape the route to human pluripotency
Source: Nat Commun. 2023 May 17;14:2829. doi: 10.1038/s41467-023-37270-w (PMC10192362; doi:10.1038/s41467-023-37270-w)
Supplement: Supplementary file 14 — Reporting Summary [file 41467_2023_37270_MOESM14_ESM.pdf]

nature portfolio

Corresponding author(s): Nicola Elvassore

Last updated by author(s): 30/01/2023

Reporting Summary

Nature Portfolio wishes to improve the reproducibility of the work that we publish. This form provides structure for consistency and transparency in reporting. For further information on Nature Portfolio policies, see our Editorial Policies and the Editorial Policy Checklist.

Statistics

For all statistical analyses, confirm that the following items are present in the figure legend, table legend, main text, or Methods section.

n/a	Confirmed	
☐	☒	The exact sample size (*n*) for each experimental group/condition, given as a discrete number and unit of measurement
☐	☒	A statement on whether measurements were taken from distinct samples or whether the same sample was measured repeatedly
☐	☒	The statistical test(s) used AND whether they are one- or two-sided<br>*Only common tests should be described solely by name; describe more complex techniques in the Methods section.*
☐	☒	A description of all covariates tested
☐	☒	A description of any assumptions or corrections, such as tests of normality and adjustment for multiple comparisons
☐	☒	A full description of the statistical parameters including central tendency (e.g. means) or other basic estimates (e.g. regression coefficient) AND variation (e.g. standard deviation) or associated estimates of uncertainty (e.g. confidence intervals)
☐	☒	For null hypothesis testing, the test statistic (e.g. *F*, *t*, *r*) with confidence intervals, effect sizes, degrees of freedom and *P* value noted<br>*Give P values as exact values whenever suitable.*
☒	☐	For Bayesian analysis, information on the choice of priors and Markov chain Monte Carlo settings
☒	☐	For hierarchical and complex designs, identification of the appropriate level for tests and full reporting of outcomes
☐	☒	Estimates of effect sizes (e.g. Cohen's *d*, Pearson's *r*), indicating how they were calculated

Our web collection on statistics for biologists contains articles on many of the points above.

Software and code

Policy information about availability of computer code

Data collection	scRNA-seq data was collected using the cellranger software (v 2.2). Fastq files were generated using the Cellranger pipeline mkfastq using 10X standard Chromium barcode sequences.
Data analysis	MS peak list files were searched by MaxQuant (v. 1.6.3.4). Principal component analysis and hierarchical clustering with heat map visualization of proteomic data was performed in MATLAB R2017a (The Mathworks). Functional enrichment analysis was performed using ReactomePA (v1.36.0) package. Reactome hierarchy was visualized using ClueGO (v2.5.6) within Cytoscape (3.8.0). Microarray data were analyzed via QuSAGE (2.26.0) package. Sequencing data was preprocessed using cellranger (v 2.2). Dimensionality reduction and trajectory inference was performed using Python (v 3.9.5) packages. Other analyses and plotting were performed in R (v 4.2.0). All the quantification reported have been performed with ImageJ software (v 2.1.0/1.53c). All statistical analyses were performed with GraphPad prism (v 7.0a). We expressed data as mean ± s.e.m or mean ± s.d of multiple biological replicates (as indicated in the figure legends).

For manuscripts utilizing custom algorithms or software that are central to the research but not yet described in published literature, software must be made available to editors and reviewers. We strongly encourage code deposition in a community repository (e.g. GitHub). See the Nature Portfolio guidelines for submitting code & software for further information.

Data

Policy information about availability of data

All manuscripts must include a data availability statement. This statement should provide the following information, where applicable:

- Accession codes, unique identifiers, or web links for publicly available datasets
- A description of any restrictions on data availability
- For clinical datasets or third party data, please ensure that the statement adheres to our policy

The authors declare that Source Data, Supplementary Datasets and Supplementary Figures are provided with this paper or from the authors upon reasonable request. The scRNA-seq raw and processed data generated in this study have been deposited in the GEO database under accession code GSE221739 [https://www.ncbi.nlm.nih.gov/geo/query/acc.cgi?acc=GSE221739]. The proteomic data generated in this study have been deposited in the Massive database under accession code MSV000090954 [https://doi.org/10.25345/C5M32NG9C]. Other databases enquired are: Protein Atlas (http://www.proteinatlas.org), Gene Ontology (http://geneontology.org/), Reactome (https://reactome.org/), MSig DB (https://www.gsea-msigdb.org/).

Human research participants

Policy information about studies involving human research participants and Sex and Gender in Research.

Reporting on sex and gender	n/a
Population characteristics	n/a
Recruitment	n/a
Ethics oversight	n/a

Note that full information on the approval of the study protocol must also be provided in the manuscript.

Field-specific reporting

Please select the one below that is the best fit for your research. If you are not sure, read the appropriate sections before making your selection.

[x] Life sciences [ ] Behavioural & social sciences [ ] Ecological, evolutionary & environmental sciences

For a reference copy of the document with all sections, see nature.com/documents/nr-reporting-summary-flat.pdf

Life sciences study design

All studies must disclose on these points even when the disclosure is negative.

Sample size	For each data shown, we performed n independent biological experiments , where n was always greater than 3. The exact n for each data also is specified in figure legends. n was chosen to be at least equal respect to typical values in the state-of-the-art.
Data exclusions	No data were excluded
Replication	All attempt at replication were successfull. Reprogramming experiments were performed independently multiple times in the timespan of multiple months.
Randomization	no relevant
Blinding	Quantifications of the imaging were blinded, therefore conducted by operators that did not know the experimental group of the images. For collection of the images no blinding was performed to avoid the exchange among experimental conditions.

Reporting for specific materials, systems and methods

We require information from authors about some types of materials, experimental systems and methods used in many studies. Here, indicate whether each material, system or method listed is relevant to your study. If you are not sure if a list item applies to your research, read the appropriate section before selecting a response.

Materials & experimental systems

n/a	Involved in the study	
☐	☒	Antibodies
☐	☒	Eukaryotic cell lines
☒	☐	Palaeontology and archaeology
☒	☐	Animals and other organisms
☒	☐	Clinical data
☒	☐	Dual use research of concern

Methods

n/a	Involved in the study	
☒	☐	ChIP-seq
☒	☐	Flow cytometry
☒	☐	MRI-based neuroimaging

Antibodies

Antibodies used	Rabbit anti-NANOG (Cell Signaling, 4903), mouse anti TRA1-60 (Millipore, MAB4360), mouse anti-STAT3 (Cell Signaling, 9139), goat anti- HGFR/c-MET (R&D, AF276). Alexa488 or Alexa594 conjugated rabbit, mouse or goat secondary antibodies (1:200) were used (Life Technologies, A21202; A21207; A11058). The nuclei were stained with Hoechst 33342 (Life Technologies).
Validation	The antibodies used are commercial antibodies, already validated in other articles. We performed positive and negative controls, and following supplier information.

Eukaryotic cell lines

Policy information about cell lines and Sex and Gender in Research

Cell line source(s)	BJ cells (Miltenyi Biotec, 130-096-726), human newborn skin fibroblasts
Authentication	The authentication of all the cell lines was provided from the supplier.
Mycoplasma contamination	Cells were periodically tested for mycoplasma contamination.
Commonly misidentified lines (See ICLAC register)	Does not apply.
